# Parental Attitudes on Social Media Monitoring for Youth: Cross-Sectional Survey Study

**DOI:** 10.2196/46365

**Published:** 2023-11-16

**Authors:** Alyssa Cohen, Anne Bendelow, Tracie Smith, Colleen Cicchetti, Matthew M Davis, Marie Heffernan

**Affiliations:** 1 Division of Advanced General Pediatrics and Primary Care, Ann & Robert H Lurie Children’s Hospital of Chicago, Chicago, IL, United States; 2 Department of Pediatrics, Northwestern University Feinberg School of Medicine, Chicago, IL, United States; 3 Mary Ann & J Milburn Smith Child Health Outcomes, Research and Evaluation Center, Stanley Manne Children’s Research Institute, Ann & Robert H Lurie Children’s Hospital of Chicago, Chicago, IL, United States; 4 Data Analytics and Reporting, Ann & Robert H Lurie Children’s Hospital of Chicago, Chicago, IL, United States; 5 Center for Childhood Resilience, Ann & Robert H Lurie Children’s Hospital of Chicago, Chicago, IL, United States; 6 Department of Psychiatry and Behavioral Sciences, Northwestern University Feinberg School of Medicine, Chicago, IL, United States; 7 Department of Medicine, Medical Social Sciences, and Preventive Medicine, Northwestern University Feinberg School of Medicine, Chicago, IL, United States

**Keywords:** social media, bullying, cyberbullying, parenting, pediatrics, well-being, wellbeing, online, monitoring, youth, internet safety, parent, survey, internet, online, mental health, cross-sectional survey

## Abstract

**Background:**

Online environments dominate the daily lives of American youth and pose evolving challenges to their health and well-being. Recent national poll data indicate that social media overuse, internet safety, and online bullying are among parents’ top child health concerns, particularly during the COVID-19 pandemic. While parents are uniquely positioned to help youth navigate social media, their attitudes on monitoring media use may be impacted by a myriad of personal and family factors.

**Objective:**

This study aimed to examine factors associated with parental attitudes about monitoring social media use among youth.

**Methods:**

Data were analyzed from the Voices of Child Health in Chicago Parent Panel Survey, administered to parents over the web and by telephone. Parents with at least 1 child aged ≥11 years responded to questions about bullying and social media monitoring from May to July 2020. The primary outcome was their response to the following question: “Do you think parents should monitor their children’s use of social media platforms such as Facebook, Twitter, and Instagram?” Bivariate analyses and multivariable logistic regression were used to examine parental agreement with frequent social media monitoring and concerns about bullying, adjusted for sociodemographic characteristics. Analyses were weighted to represent the parent population of Chicago.

**Results:**

Among 1613 survey respondents, the analyzed sample included 808 parents with at least 1 child aged ≥11 years. Overall, 62.9% (n=566) of parents agreed with frequent parental monitoring of their children’s social media use. Compared with parents aged ≤35 years, parents who were >35 years old were significantly less likely to agree with frequent social media monitoring (adjusted odds ratio [aOR] 0.45, 95% CI 0.25-0.81). Parents expressing a high level of concern regarding the effects of bullying were more likely to agree with frequent monitoring of youth social media (aOR 2.15, 95% CI 1.24-3.73).

**Conclusions:**

Parents’ personal characteristics and concerns about bullying may influence their attitudes toward monitoring social media use among youth. Given the potential impact of these attitudes on parental monitoring behaviors and the subsequent health impact on youth, pediatricians should consider these factors when counseling about bullying and social media. Child health professionals can support families in developing a safe media use plan that fits family circumstances.

## Introduction

Online environments dominate the lives of American youth and pose evolving challenges to their health and well-being. The overwhelming majority of adolescents have smartphone access and almost half report being online “almost constantly” [1]. Social media represents an important proportion of youth’s internet use, providing novel and rapidly shifting platforms for communication and content-sharing [2,3]. While social media may positively impact youth by facilitating social connection [4] and providing resources for seeking support [5], there are numerous potential health risks associated with online platforms, including sleep disruption [6], problematic internet use [7], and cyberbullying [8-10]. Cyberbullying, defined as the use of electronic communication technologies to bully others [11,12], is widespread among youth, with 59% of US teens reporting that they have experienced online victimization [13].

The impact of online media is also felt by parents. In a recent national poll [14], parents rated overuse of social media, cyberbullying, and internet safety among their top child health concerns. Parents are uniquely positioned to help youth navigate online environments. The extant literature demonstrates that parental monitoring and involvement in media use can mitigate associated risky health behaviors and reduce cyberbullying [15-17]. However, much remains unknown about factors that impact parental monitoring behaviors. For pediatricians and child advocates, who may support families in navigating online media use, it is important to take these factors into consideration.

Drawing on the theory of planned behavior, which maintains attitudes as core determinants of human social behavior [18], we posit that an improved understanding of parental attitudes around social media and cyberbullying may be key to impacting their approach to monitoring youth’s online activities. Using parent survey data, this study sought to characterize parental attitudes on social media monitoring for youth. We hypothesized that parental agreement with frequent social media monitoring is related to parents’ personal characteristics and their concerns about bullying.

## Methods

### Ethical Considerations

The institutional review board at Ann & Robert H Lurie Children’s Hospital of Chicago determined this study to be exempt (IRB 2019-3063) and that all methods were in accordance with ethical standards. The institutional review board at NORC (National Opinion Research Center) also approved all study activities.

### Study Design

Participants were first recruited from the probability-based Voices of Child Health in Chicago (VOCHIC) panel and NORC’s AmeriSpeak panel with a 66.2% response rate (1035 responses from 1564 eligible invitees). To ensure a sufficient sample size, the probability sample was augmented by calibration-weighted, non–probability-based responses through opt-in, web-based panels (n=578). The survey was administered by NORC via the web and telephone from May to July 2020 (n=1613) (see additional details in Multimedia Appendix 1 [19-21]). Eligibility criteria included age ≥18 years, being the parent of at least 1 household child, and Chicago residence [19-21]. The present study focused on a subgroup of parents with at least 1 child aged ≥11 years (n=808) to capture parents of children most likely to be active users of social media. Respondents were compensated US $10 for survey completion.

### Measures

All measures included in this analysis were part of a broader survey of social emotional learning among children and adolescents, obtained during the VOCHIC summer 2020 administration.

#### Dependent Variables

The primary outcome measure was the response to the question, “Do you think parents should monitor their children’s use of social media platforms such as Facebook, Twitter, and Instagram?” Based on sample response distribution, and to facilitate analyses with a focus on frequent social media monitoring as best aligned with existing recommendations for parental monitoring [22], response options of “yes, frequently,” “yes, some of the time,” and “no” were collapsed to create a dichotomous variable of “yes, frequently” and “some of the time or no.”

#### Independent Variables

We explored demographic variables and parents’ concerns about bullying as potential predictors. Household income was combined into 3 groups based on the US federal poverty level (FPL; <100% FPL, 100%-399% FPL, ≥400% FPL) [23]. Parent education (high school education or below, some college, college degree or higher) and parent age (18-35 years, ≥36 years with midpoint based on subsample distribution) were also assessed. Parents’ self-reported race and ethnicity were combined into 4 groups (Black, White, Latinx, and Asian/Other race). Race and ethnicity were included in the analyses to investigate previously reported differences in parental concerns regarding cyberbullying and internet safety [12], as well as differences in youth internet use [1], among minoritized racial and ethnic groups. We examined the age of the oldest child in the household using a dichotomized variable (11-13 or 14-17 years old). The cutoff at 13-14 years was selected to differentiate younger adolescents and preadolescents from their high school–aged counterparts, who may demonstrate different social media use patterns. Parent gender was assessed as male, female, or nonbinary. Due to the small subsample size of nonbinary parents (n=3), this group was omitted from gender analyses. Child school type was coded as “public,” “private/charter,” or “other.”

We assessed parents’ level of concern about bullying with the item, “How concerned are you about the following?” followed by 4 bullying concerns compiled as a composite variable, as outlined in Textbox 1.

Textbox 1. **Survey items and composite variable related to parental concerns about bullying.**

**Parental concerns**

1. Long-term effects of bullying, such as effects that last into adulthood

2. Short-term effects of bullying, such as kids feeling left out

3. Physical harm or injury due to bullying

4. Mental health or psychological effects of bullying

**Response options**

1. Not at all concerned

2. Not very concerned

3. Somewhat concerned

4. Very concerned

5. Extremely concerned

**Composite variable**

1. Low bullying concern

- 0: “very” or “extremely” concerned responses

2. Medium bullying concern

- 1-3: “very” or “extremely” concerned responses

3. High bullying concern

- 4: “very” or “extremely” concerned responses

### Statistical Analysis

Bivariate analyses compared survey responses across sociodemographic characteristics and bullying concern categories. *χ*^2^ tests determined the association between predictors and parental agreement with frequent social media monitoring. A multivariable logistic regression model included statistically significant (*P*<.05) predictors from bivariate analyses. All statistical analyses were population weighted (see Multimedia Appendix 1), used a significance level of *P*<.05, and were performed using SAS software (version 9.4; SAS Institute).

## Results

Of the 808 parent respondents with at least 1 child aged ≥11 years, 566 (62.9%) agreed that parents should frequently monitor their children’s social media. Only 30 (5.2%) parents responded “no” to the primary outcome question, with the remainder responding “yes, some of the time” (n=212, 31.9%). For all other analyses, responses to the primary outcome were dichotomized as described in the *Methods* section. Bivariate analyses demonstrated significant differences (*P*s<.05) in response to frequent social media monitoring across parent race/ethnicity, parent and oldest child age, parental education, child school type, and bullying concern level, but not household income or parent gender (Table 1).

**Table 1.** Sociodemographic characteristics of the survey sample, by parental agreement with frequent social media monitoring for youth.

		Responses^a^		*P* value^b^
		“Yes, frequently,” n (%)^c^	“Some of the time” or “no,” n (%)	
Survey sample		566 (62.9)	242 (37.1)	—^d^
**Parent race/ethnicity (n=808**)				<.001
	White, non-Hispanic/Latinx	208 (63.3)	79 (36.7)	
	Black, non-Hispanic/Latinx	147 (77.2)	45 (22.8)	
	Hispanic/Latinx	188 (61.5)	92 (38.5)	
	Asian/Other^e^, non-Hispanic/Latinx	23 (32.5)	26 (67.5)	
**Parent age (n=808)**				.009
	≤35 years	137 (74.1)	53 (25.9)	
	>35 years	429 (60.1)	189 (39.9)	
**Household income (n=790)**				.77
	<100% FPL^f^	99 (59.2)	38 (40.8)	
	100%-399% FPL	227 (63.5)	103 (36.5)	
	≥400% FPL	227 (63.5)	96 (36.5)	
**Parental education (n=802)**				.03
	High school or less	122 (58.4)	71 (41.6)	
	Some college or technical school	149 (74.2)	46 (25.8)	
	College degree or higher	291 (62.2)	123 (37.8)	
**Parent gender (n=803)**				.08
	Male	170 (58.0)	91 (42.0)	
	Female	394 (67.1)	148 (32.9)	
**Oldest child age (n=808)**				.01
	11-13 years	251 (71.8)	62 (28.2)	
	14-17 years	315 (58.4)	180 (41.6)	
**Child school type (n=762)^g^**				.04
	Private/charter	188 (71.5)	65 (28.5)	
	Public	353 (60.6)	156 (39.4)	
**Bullying concern level (n=797)**				.005
	Low	123 (53.5)	74 (46.5)	
	Medium	189 (61.0)	85 (39.0)	
	High	247 (72.8)	79 (27.2)	

a Responses to the survey item “Do you think parents should monitor their children’s use of social media platforms such as Facebook, Twitter, and Instagram?”

b A significance level of *P*<.05 was used as the threshold for variable inclusion in the multivariable analysis.

c Survey-weighted frequencies are displayed as percentages in rows; number (n) of participants are displayed as unweighted counts.

d Not applicable.

e The Asian/Other race group included non-Hispanic/Latinx groups self-identifying as Chinese, Filipino, Japanese, Korean, Vietnamese, Asian Indian, Samoan, Guamanian or Chamorro, Native Hawaiian, Other Pacific Islander, American Indian or Alaska Native, and “Some other race.”

f FPL: federal poverty level.

g Child school type was coded as “public” if all children aged 11-17 years attended public school, “private/charter” if all children aged 11-17 years attended private or charter school, and “other” if children attended another type of school or if children in the household attended different types of schools. The “other” group was omitted from analyses due to heterogeneity and small sample size.

In multivariable logistic regression, parents over age 35 had significantly lower odds of agreeing with frequent social media monitoring for youth (adjusted odds ratio [aOR] 0.45, 95% CI 0.25-0.81), compared with younger parents (Table 2).

**Table 2.** Adjusted odds ratio (aOR) of parental agreement with frequent social media monitoring for youth, by sociodemographic characteristics and bullying concerns.^a^

		aOR	95% CI	*P* value
**Parent race/ethnicity (ref^b^: White, non-Hispanic/Latinx)**				
	Black, non-Hispanic/Latinx	1.85	0.92-3.73	.08
	Hispanic/Latinx	0.97	0.51-1.83	.92
	Asian/Other, non-Hispanic/Latinx	0.34	0.14-0.82	.02
**Parent age (ref: ≤35 years)**				
	>35 years	0.45	0.25-0.81	.007
**Parental education (ref: high school or less)**				
	Some college or technical school	1.88	1.03-3.43	.04
	College degree or higher	1.11	0.61-2.00	.74
**Parent gender (ref: male)**				
	Female	1.12	0.68-1.84	.65
**Oldest child age (ref: 14-17 years)**				
	11-13 years	1.57	0.94-2.62	.08
**Child school type (ref: all children in public)**				
	All children in private/charter	1.47	0.86-2.49	.16
**Bullying concern level (ref: low)**				
	Medium	1.72	0.98-3.02	.06
	High	2.15	1.24-3.73	.007

a Multivariable regression model adjusted for all variables listed in the table.

b Ref: reference.

Respondents who identified as Asian/Other race also had lower odds of agreement with frequent social media monitoring (aOR 0.34, 95% CI 0.14-0.82) than White parents. Parents had higher odds of agreement with frequent monitoring if they had some college or technical school education (aOR 1.88, 95% CI 1.03-3.43), compared with high school or less. Having a high level of concern about bullying was associated with 2.15 times increased odds of agreement with frequent social media monitoring among parents (95% CI 1.24-3.73), compared with a low level of bullying concern. In adjusted analyses, parent gender, child age, and child school type were no longer associated with agreement with social media monitoring.

## Discussion

### Principal Findings

In a representative sample of parents in a large, socioeconomically diverse, urban population, we found that most parents agreed with frequent monitoring of social media use among youth. Parents were more likely to have positive attitudes about frequent social media monitoring if they were younger and more concerned about the negative effects of bullying. As attitudes are a key determinant of behavior, these findings add important context to the literature surrounding the monitoring of media use in youth, amid mounting parental concerns regarding online environments and child health [14].

In addition to personal attitudes, recent studies have suggested that elements such as knowledge, perceived control, and risk assessment may be key to understanding parents’ involvement in youth social media and cyberbullying prevention [24,25]. Indeed, our results indicated that younger parents, who might have more personal familiarity with social media than older parents, were more likely to agree with frequent monitoring of youth. In discussing the monitoring of youth media use, it is important for pediatricians to consider how parents’ own experiences and familiarity with various platforms may impact their attitudes and, therefore, behavior.

Our findings also highlight that concerns about the risks of bullying may be an important driver of parents’ attitudes on social media monitoring. For pediatricians who routinely provide guidance around bullying, these conversations may afford an ideal opportunity to also explore families’ media use practices and address concerns about online environments. Child health professionals are well positioned to provide education and make recommendations, including on the development of a family media plan and active supervision of youths’ online activities [11].

### Limitations

It is important to acknowledge this study’s limitations. While results are weighted to represent households in Chicago, they may not generalize to other populations. However, Chicago is a diverse city with similar demographics to the United States more broadly, so we expect that results likely generalize to other large US cities [26]. Importantly, respondents were voluntary members of a panel and may respond differently than parents in the general population. Responses to the primary outcome variable were collapsed from 3 into 2 options based on subsample sizes and alignment with recommendations for parental monitoring practices, and may not fully capture nuanced differences among participants’ attitudes. Similarly, some demographic data, such as parent age, were measured categorically, limiting analysis options. The subsample of participants identifying as Asian/Other race/ethnicity was small and heterogeneous, limiting the interpretability of results for this group. Finally, the effect of nonresponse bias should be considered given the study’s survey-based design.

### Conclusions

Amid mounting parental concerns regarding online media, this large study of a diverse group of urban parents indicates that attitudes regarding monitoring of youth social media use vary. Personal characteristics such as parent age and concern for the health impact of bullying were associated with parents’ agreement with frequent social media monitoring for youth. These factors may provide helpful context for pediatricians as they support families in navigating safe media use. Improved understanding of parents’ attitudes about social media will continue to be an essential focus as future research examines targets for intervention to promote healthy social media use among youth.

## Supplementary material

10.2196/46365 Multimedia Appendix 1 Supplemental information on methods.

## Abbreviations

aOR: adjusted odds ratio
FPL: federal poverty level
NORC: National Opinion Research Center
VOCHIC: Voices of Child Health in Chicago
