# Supplementary material for: Parental Attitudes on Social Media Monitoring for Youth: Cross-Sectional Survey Study
Source: JMIR Pediatr Parent. 2023 Nov 16;6:e46365. doi: 10.2196/46365 (PMC10670659; doi:10.2196/46365)
Supplement: Multimedia Appendix 1 [file pediatrics-v6-e46365-s001.docx]

**Supplement for Methods**

**Voices of Child Health in Chicago – Parent Panel Survey**

The Voices of Child Health in Chicago (VOCHIC) Parent Panel Survey is a triannual survey of Chicago parents about child, adolescent, and family health and well-being. Parents in the panel are from all 77 neighborhoods in Chicago. One parent per household could complete the survey online or over the phone. Web-based surveys have increased in popularity over the last decade as phone survey response rates have declined [19]. Respondents provided consent online or over the phone, depending on their survey mode.

**Recruitment and Sampling Methodology**

The current used data from the first wave of the VOCHIC Parent Panel. Parent respondents were recruited to participate in this wave through one of three mechanisms implemented by NORC at the University of Chicago: 1) address-based sampling (ABS) with mailed recruitment materials, 2) NORC’s probability-based AmeriSpeak panel, and 3) established online nonprobability survey panels (Dynata and Lucid panels). Responses from nonprobability samples were included to ensure sufficient sample size, which has been shown to be a cost-effective method to supplement probability-based samples [20]. The overall response rate for the probability-based respondents (ABS and AmeriSpeak sample) was 66.2% (1,035 out of 1,564 invitees). A response rate (i.e., denominator) for the non-probability sample was unable to be measured because these panels administer opt-in online surveys. Probability-based respondents were compensated $10 for completing the survey. Non-probability respondents were compensated by their home panel.

**Data Weighting and Analysis**

For the probability-based samples, base sampling weights are adjusted to account for nonresponse via a raking ratio method to American Community Survey (ACS) 18+ Chicago parents population totals associated with the following topline socio-demographic characteristics: age, sex, education, race/ethnicity, and Census Division, and the following socio-demographic interactions: age × gender, age × race/ethnicity, and race/ethnicity × gender.

For the nonprobability samples, we explicitly account for potential bias using NORC’s True North calibration [21], a hybrid calibration approach developed at NORC based on small area estimation methods. The purpose of TrueNorth calibration is to adjust the weights for the nonprobability sample to bring weighted distributions of the nonprobability sample in line with the population distribution for characteristics correlated with the survey variables. Such calibration adjustments help to reduce potential bias, yielding more accurate population estimates.
